# Comatulids (Crinoidea, Comatulida) chemically defend against coral fish by themselves, without assistance from their symbionts

**DOI:** 10.1038/s41598-020-63140-2

**Published:** 2020-04-09

**Authors:** Alexander Kasumyan, Olga Isaeva, Polina Dgebuadze, Elena Mekhova, Le Thi Kieu Oanh, Temir Britayev

**Affiliations:** 1 Department of Ichthyology, Faculty of Biology, Lomonosov Moscow State University, Leninskiye Gory 1, page 12, 119234 Moscow, Russia; 2 Severtsov Institute of Ecology and Evolution, Leninskiy pr. 33, 117071 Moscow, Russia; 3 Department of Water Bioresources and Aquaculture, Kamchatka State Technical University, Vilyuiskaya st. 56, 683001 Petropavlovsk-Kamchatsky, Russia; 4 Russian-Vietnamese Tropical Research and Technological Center, Coastal Branch, Nguyen Thien Thuat 30, Nha Trang, Vietnam

**Keywords:** Tropical ecology, Animal behaviour

## Abstract

Symbiotic associations between small animals and relatively large sessile invertebrates that use taste deterrents for protection are widespread in the marine environment. To determine whether the symbionts are involved in the chemical protection of their hosts, the palatability of ten species of comatulids and six species of their symbionts was evaluated. Taste attractiveness was determined by offering agar pellets flavoured with extracts of comatulids and their symbionts for four coral reef fish species. Five species of symbiont were highly palatable, and one was indifferent to the taste. Almost all comatulids were distasteful, while their aversiveness was different for different fish. These findings indicate that comatulids chemically defend themselves without assistance from symbionts, and the taste deterrents are not universal and can only be effective against particular predators. The presence of tasteful symbionts reduces the security of their hosts by provoking attacks of predators and may impact on the individual and population fitness of comatulids. However, the chemical protection of comatulids is useful for symbionts and undoubtedly increases their survival. Obtained results allows the relationship between comatulids and their symbionts considered commensalism. Most likely, similar relationships can be established in many other associations, where symbionts inhabit chemically defended coral reef invertebrates.

## Introduction

In coral communities, which are characterized by extremely high species diversity and an unusually wide range of biotic relationships among organisms, the symbiotic coexistence of species is very common^1–3^. At least 860 symbionts were recorded in only one host group, the scleractinian corals^4^. A comparable diversity of symbionts is associated with other host groups of coral reef animals, comatulids or feather stars (Сrinoidea, Comatulida)^5–14^.

Comatulids are sessile animals with limited mobility. To reduce their vulnerability to predators, they have adopted various morphological, behavioural, chemical and other protective adaptations. These may include body rigidity, dense crown, cryptic mode of life, fast regeneration, aversive taste, and toxicity^15–18^. Based on field and aquaria observations, it has been assumed that fish avoid feeding on comatulids^19^. Later, the repulsive taste of some comatulids for several fish species was confirmed experimentally^20,21^. There are no fish species specialized in feeding on comatulids; nevertheless, sometimes fish attack them, and findings of comatulid fragments in the digestive tracts of some fishes are well documented^19,22–24^.

Diverse symbiotic communities comprising tens of species have been found in all studied comatulids on the coral reefs of South Vietnam^25,26^. Among their symbionts are polychaetes, gastropods, crabs, shrimps, brittle stars and fish. The total number of symbiotic species recorded just in one bay was close to seventy^25^. Up to ten species and more than fifty individuals of symbionts can inhabit one host specimen simultaneously^13^.

The symbionts inhabiting comatulids can affect the vulnerability of their hosts. However, the nature of the relationship between comatulids and their diverse symbionts remains unclear. Special studies on this issue are absent; however, it has been suggested that symbionts, hiding in the crowns of comatulids, attract predators, especially fish, to their hosts^27^. Hunting on symbiont fish can injure their hosts during attacks^27–30^. This was considered as the main reason for the frequent injuries of comatulid arms and piunnules^28–30^.

The benefit of symbionts depends on the hosts’ fitness and sustainability, and in some cases, their contribution to the protection of hosts is obvious^31–37^. The number of described symbiotic associations with mutually beneficial types of interactions is rapidly increasing^38^. It is known that some symbionts are able to defend their hosts from predators. *In situ* experiments have shown that artificial removal or natural loss of symbionts can lead to rapid extermination of their host^39,40^. Symbionts of comatulids are not large enough to offer their hosts active protection against predators. However, being deterrent, they can enhance the host’s chemical defence. In contrast, if the symbionts of the comatulids have an attractive taste, their presence may increase the risk of predator attacks on the host. Studies investigating the chemosensory characteristics of comatulid’s symbionts for fish and other consumers may support one of these hypotheses, but as yet, these studies have not been performed. The possible chemical protection of symbionts based on the use of taste deterrents remains unknown.

Therefore, the main goal of the present study was to assess the taste attractiveness of comatulid symbionts for fishes and to compare it with that of their hosts. To this end, the palatabilities of artificial pellets flavoured with water extract of symbionts and their comatulid hosts were evaluated for omnivorous coral reef fish in a series of laboratory experiments. We used water extraction because it is natural way for taste substances to be released from food and to reach the fish oral taste receptors. The use of ten feather star species and four fish species in the course of the experiments made it possible to assess the variability of the reactions of different fish species to the taste deterrents of the same species of feather stars.

## Results

### Symbionts

**Figure 1:**
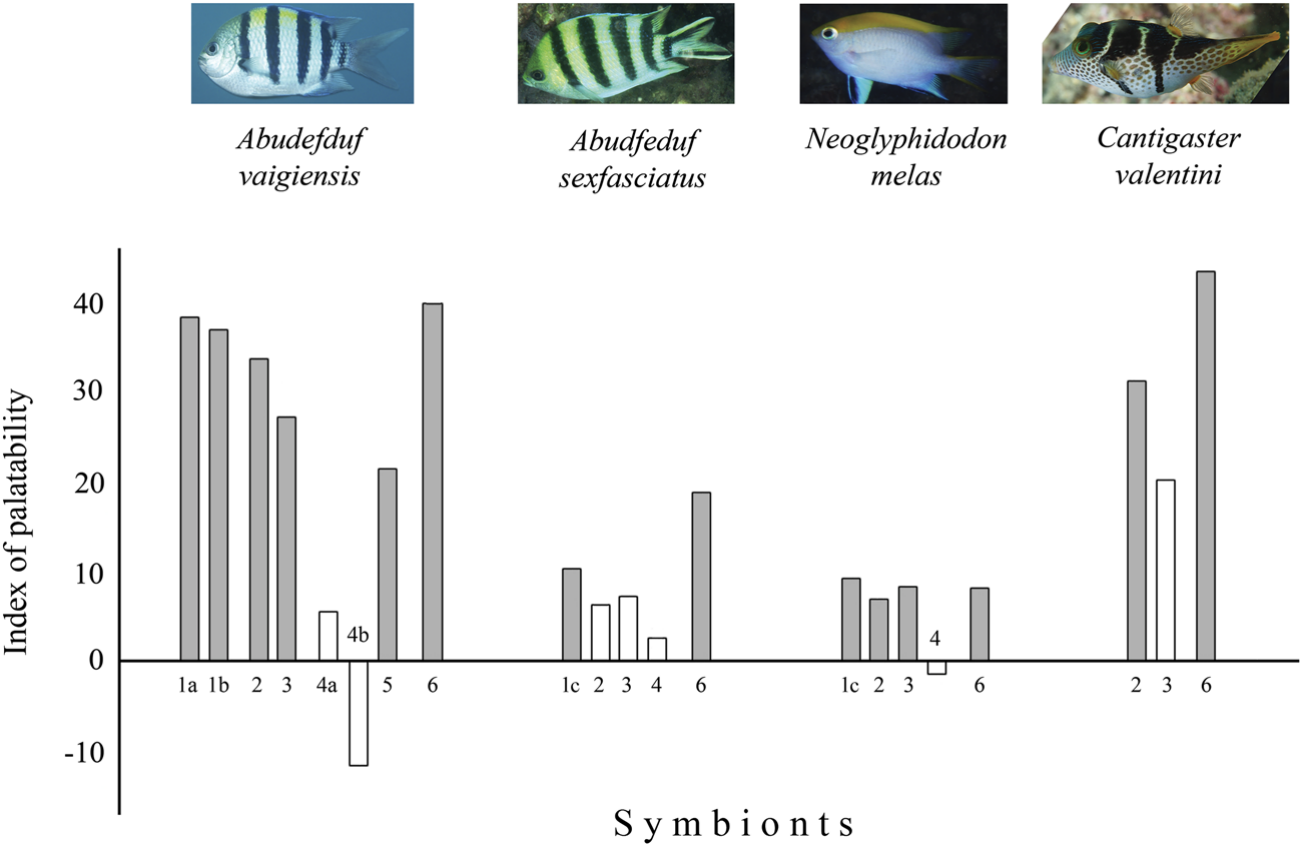
The taste attractiveness of comatulid symbionts for fish. Tested species numbers: (**a**) *Synalpheus stimpsoni*; (**b**) *Synalpheus demani*; (**c**) *Synalpheus* sp.; 2 – *Allogalathea elegans;* 3 *– Paradyte crinoidicola;* 4 *– Gymnolophus obscura;* 4a and 4b *– Gymnolophus obscura* collected on *Comaster nobilis* and *Himerometra robustipinna*, respectively; 5 – *Notopharyngoides aruensis*; and 6 *– Litopenaeus vannamei*. Shaded bars – the significant difference (P < 0.05) in consumption of flavoured pellets in relation to the control Photo courtesy of O.V. Savinkin and Yu.V. Deart.

The flavouring of agar gel with an extract of symbionts significantly increases the consumption of pellets in most cases. In two fish, *Neoglyphidodon melas* and *Abudefduf vaigiensis*, such an effect was caused by extracts of all symbionts. The efficiency of extracts was comparable with that of the shrimp *Penaeus vannamei*, which is used for daily fish feeding. The exception was the ophiuroid *Gymnolophus obscura*, which had an inert taste regardless of what comatulids, *Comaster nobilis* or *Himerometra robustipinna*, they were collected from (Table 1, Fig. 1). For the fish *Abudefduf sexfasciatus*, only the shrimp Synalpheus sp. has a significantly attractive taste. Extracts of the galatheid *Allogalathea elegans* and the polychaete *Paradyte crinoidicola* increase the consumption of pellets, but due to the high consumption of control pellets (81.3%), the results of the experiments were not significant. Extract of ophiuroid also has an inert taste for this fish. For *Canthigaster valentini*, only the extract of *Allogalathea elegans* was palatable.

**Table 1:** Consumption of agar pellets flavoured with water extract of comatulids and their symbionts by four fish species. Values are presented as *M* ± *m* – mean and standard error of mean. The concentrations of water extracts are 300 mg/ml for all species except *Allogalathea elegans* and *Paradyte crinoidicola* (150 mg/ml) and *Notopharyngoides aruensis* (116 mg/ml) in trials on *Abudefduf vaigiensis*. *, ** and *** – differences from the control are significant at P < 0.05, P < 0.01 and P < 0.001, respectively. Cr – Crustacea, M – Myzostomida, P – Polychaeta, O – Ophiuroidea. ^§^ – host comatulid species not specified. # – species of *Synalpheus* not identified.

Species	*Abudefduf vaigiensis*		*Abudefduf sexfasciatus*		*Neoglyphidodon melas*		*Cantigaster valentini*	
	Pellets consumption, %	n	Pellets consumption, %	n	Pellets consumption, %	n	Pellets consumption, %	n
**Comatulids:**								
*Comaster nobilis*	48.0 ± 14.3	50	57.1 ± 0.1*	63	43.5 ± 6.0***	69	—	
*Clarkcomanthus alternans*	36.8 ± 12.9	57	64.5 ± 0.1	62	45.7 ± 6.0***	70	—	
*Stephanometra indica*	23.3 ± 11.0*	60	56.5 ± 0.1*	62	4.7 ± 2.7***	64	—	
*Anneissia pinguis*	24.1 ± 11.8*	54	62.9 ± 0.1*	62	25.9 ± 6.0***	54	—	
*Comanthus gisleni*	21.7 ± 10.7*	60	54.8 ± 0.1**	62	15.4 ± 5.1***	52	—	
*Himerometra robustipinna*	15.0 ± 9.3**	60	30.6 ± 0.1***	62	34.8 ± 5.8***	69	—	
*Cenometra bella*	8.3 ± 7.2***	60	59.7 ± 0.1*	62	5.4 ± 3.0***	56	—	
*Comanthus parvicirrus*	0.0 ± 0.0***	52	1.6 ± 0.0***	62	10.2 ± 4.0***	59	—	
*Colobometra perspinosa*	—		59.0 ± 0.1*	61	11.6 ± 3.9***	69	—	
*Lamprometra palmata*	—		66.1 ± 0.1	62	4.2 ± 2.4***	71	—	
Control	—		76.9 ± 0.1	65	—			
**Symbionts:**								
*Allogalathea elegans* (Cr)	98.8 ± 1.2***	84	92.2 ± 0.0	51	94.6 ± 2.6**	74	65.5 ± 9.0*	29
*Paradyte crinoidicola* (P)	85.7 ± 3.8***	84	94.0 ± 0.0	50	97.3 ± 1.9**	73	51.7 ± 9.4	29
Control	49.2 ± 4.5	126	—		—		—	
*Synalpheus* sp.# (Cr)	—		100 ± 0.0**	51	98.6 ± 1.4***	73	—	
*Synalpheus stimpsoni* (Cr)	96.7 ± 4.7***	60	—		—		—	
*Synalpheus demani* (Cr)	93.3 ± 6.5***	60	—		—		—	
*Paradyte crinoidicola* (P)	85.7 ± 3.8***	84	94.0 ± 0.0	50	97.3 ± 1.9**	73	—	
*Gymnolophus obscura*^§^ (O)	—		85.7 ± 0.1	49	81.7 ± 4.6	71	—	
*Gymnolophus obscura* (O):								
host *H. robustipinna*	48.3 ± 13.0	60	—		—		—	
host *C. nobilis*	34.2 ± 11.1	73	—		—		—	
*Notopharyngoides aruensis* (M)	66.7 ± 12.3**	60	—		—		—	
Control	43.2 ± 7.1	190	81.3 ± 0.1	48	—		—	
*Penaeus vannamei* (Cr)	100.0 ± 0.0***	140	100 ± 0.0***	77	97.0 ± 1.7***	99	86.8 ± 5.6***	38
Control	43.2 ± 7.1	190	77.5 ± 0.0	89	82.4 ± 2.2	290	34.4 ± 8.5	32

### Comatulids

**Figure 2:**
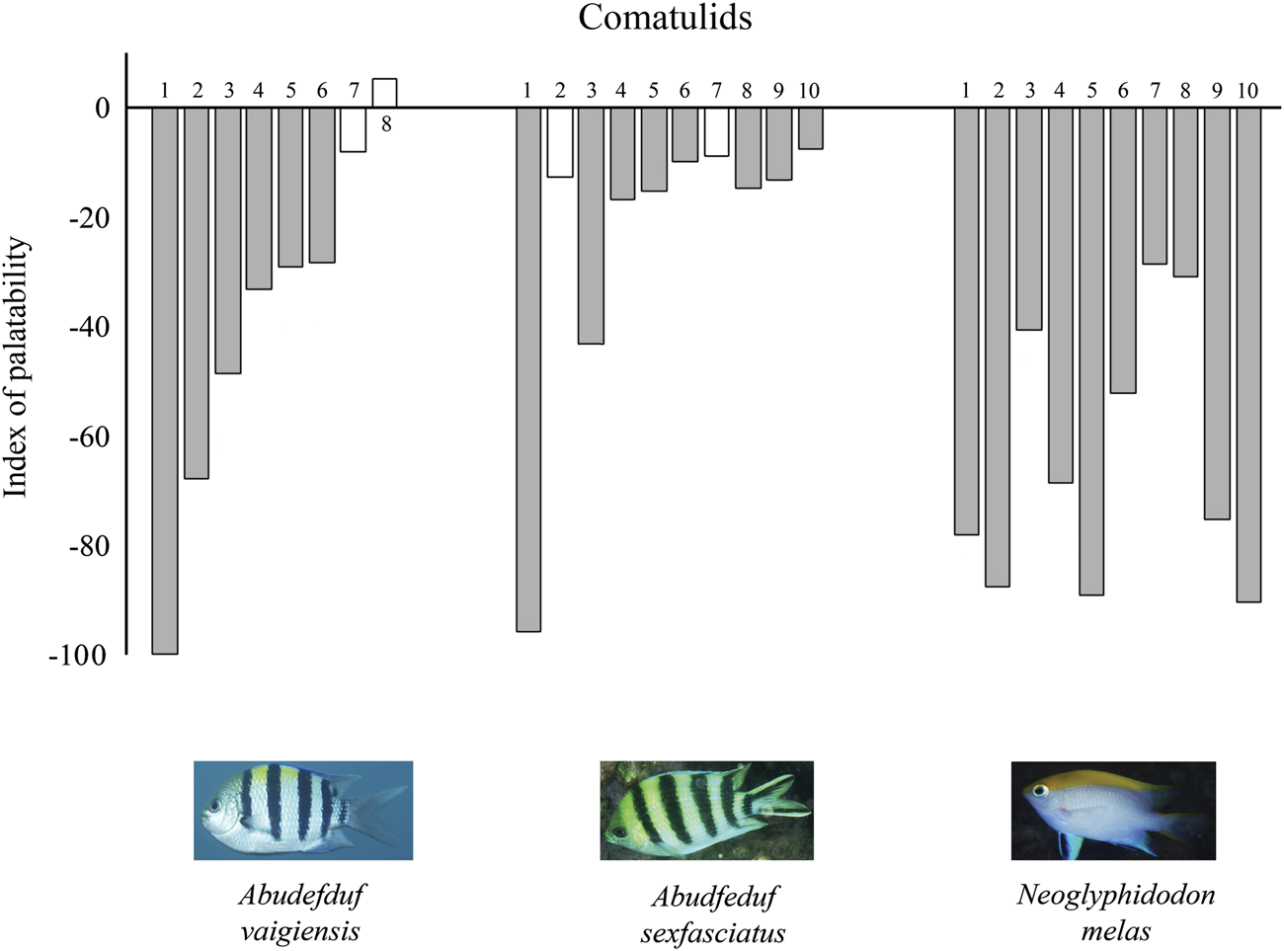
The taste attractiveness of comatulids for fish. Tested species numbers: 1 – *Comaster nobilis;* 2 – *Clarckomanthus alternans;* 3 – *Stephanometra indica;* 4 – *Anneissia pinguis;* 5 – *Comanthus gisleni;* 6 – *Himerometra robustipinna;* 7 – *Cenometra bella;* 8 – *Comanthus parvicirrus;* 9 – *Colobometra perspinosa;* and 10 – *Lamprometra palmata*. Shaded bars – the significant difference (P < 0.05) in consumption of flavoured pellets in relation to the control Photo courtesy of O.V. Savinkin and Yu.V. Deart.

Pellets containing comatulid extract were either consumed less frequently than the control pellets or provoked a neutral response. None of the extracts leads to an increased consumption of pellets in any of three fish species. The most negative attitude towards the taste of comatulids was observed in *Neoglyphidodon melas*: extracts of all ten comatulid species caused significant decreases in pellet consumption (p < 0.001 for all). Extracts of *Stephanometra indica, Cenometra bella* and *Lamprometra palmata* had the strongest aversive effect, decreasing the consumption of pellets by 15–20 times relative to the control. *Clarckomanthus alternans, Comaster nobilis* and *Himerometra robustipinna* extracts showed the weakest effect, reducing the consumption of pellets by approximately 2 times. For *Abudefduf vaigiensis* extracts of six species of comatulids from eight tested evoked an aversive effect; of them, *Cenometra bella* extracts (>5 times) and *Comanthus parvicirrus* (complete blocking of consumption) most strongly inhibited pellet consumption. For *Abudefduf sexfasciatus* extracts of eight species of comatulids from ten tested were distasteful. The extract of one of them, *Comanthus parvicirrus*, caused a decrease in pellet consumption by almost 50 times relative to the control (Table 1, Fig. 2).

**Figure 3:**
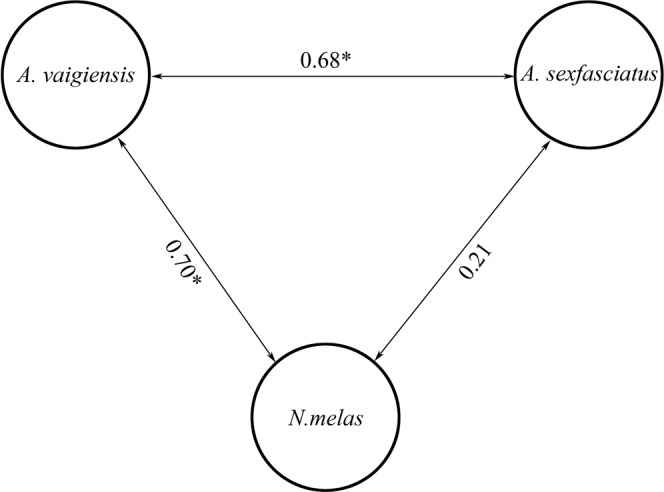
Spearman rank correlations between taste attractiveness of agar pellets flavoured with extracts of comatulids for *Abudefduf vaigiensis, Neoglyphidodon melas* and *Abudefduf sexfasciatus*.

A significant similarity between the palatability of agar pellets flavoured with extracts of comatulids was revealed between *Abudefduf vaigiensis* and two other fish species, *Abudefduf sexfasciatus* (*r*_*s*_ = 0.68; p < 0.05) and *Neoglyphidodon melas* (*r*_*s*_ = 0.70; p < 0.05) but not between *Neoglyphidodon melas* and *Abudefduf sexfasciatus* (*r*_*s*_ = 0.21; p > 0.05) (Fig. 3).

## Discussion

Our study is the first to evaluate palatability for fish of different symbiotic species associated with comatulids. The results clearly demonstrate that comatulids and their symbionts have opposite taste properties for fish. Most of the studied symbionts (five from six species) are highly palatable, in contrast to comatulids, of which almost all species are distasteful. The deterrence of comatulids and the palatability of inhabiting them symbionts suggest that symbionts do not take part in the chemical defence of their host. Quite the contrary, our data prove the hypothesis that the presence of symbionts, the desired prey for fish, reduces the security of their hosts by provoking attacks of predators on comatulids. Most likely, it is the fishes’ hunt for symbionts, and not for their hosts, that leads to the appearance of injuries on the arms of the comatulids^27–29^. However, the chemical protection of comatulids is undoubtedly useful for symbionts and increases their survival.

### Symbionts

Symbionts palatable to fish belong to two systematic groups: Crustacea (shrimps from the genus *Synalpheus* and the galatheid *Allogalathea elegans*) and Polychaeta (the scaleworm *Paradyte crinoidicola* and the mizostomid *Notopharyngoides aruensis*). Shrimps of the genus *Synalpheus* have the most pronounced stimulating effect for all three fish species on which these trials were conducted (*Neoglyphidodon melas* and two *Abudefduf* species). Attractive taste is inherent in most previously studied crustaceans, and species with aversive taste are rare among them, while among polychaetes, species with taste that is unpleasant for fish are more common^41–47^.

Of the symbionts studied, the ophiuroid *Gymnolophus obscura* has the least palatability; its taste for all tested fishes is indifferent. The taste quality of *Gymnolophus obscura* does not depend on the palatability of its host. An experiment performed on *Abudefduf vaigiensis* showed that the ophiuroid associated with neutral in taste *Comaster nobilis* does not differ in palatability from the ophiuroid collected from the deterrent *Himerometra robustipinna* (Fig. 1).

It should be emphasized that many ophiuroids and other echinoderms studied to date, including starfishes, holothurians and comatulids, use chemical deterrents to acquire aversive taste as protection from predators^20,48–56^. It can be assumed that the ancestral free-living forms of *Gymnolophus obscura* were also chemically defended, but later, having switched to a symbiotic mode of life, they gradually lost this characteristic, which required energy expenses^57^ but became useless for them. *Gymnolophus obscura* living on the oral disc of comatulids is well hidden from potential predators, despite its relatively large size. *G. obscura*’s secondary acquisition of a taste indifferent to fish seems to be a quite plausible scenario of possible evolutionary transformations of this species.

### Comatulids

Until recently, information about the taste of comatulids for fish was controversial. Both damages to comatulids, which were associated with attacks by fish, and the presence fragments of comatulids in the digestive tracts of fish, indirectly prove the absence of taste deterrents in the comatulids^22,28,29,58^. At the same time, the high abundance and diversity of comatulids in coral reefs, where the density of fish is extremely high, suggest the use of deterrent taste for survival^59^. In recent years, the first experimental evidence of the presence of taste deterrents in comatulids, making them unpalatable for fish, their most potential predators, have appeared^20,21,60^. Our study strongly confirm these data: all ten studied comatulids are distasteful for *Neoglyphidodon melas*, six out of eight species for *Abudefduf vaigiensis*, and eight out of ten species for *Abudefduf sexfasciatus*. Notably, comatulids with a taste attractive for fish were not found.

The taste deterrence of comatulid species is not the same and varies from low to high degrees. For example, the difference exceeded ten-fold if comparing consumption pellets flavoured with extracts of highly distasteful *Lamprometra palmata*, *Stephanometra indica* and *Cenometra bella* and extracts of *Comaster nobilis* and *Clarkcomanthus alternans*, with lower taste aversiveness for *Neoglyphidodon melas*. The question of whether difference in deterrence between comatulids depends on different chemical substances, their different proportions or different concentrations remains completely open. As has been reported earlier^60^, polyketide sulphates, which are polycarbonyl secondary metabolites, could be one of these substances.

Comatulids have also been shown to differ in their palatability between fish species. For example, *Lamprometra palmata* was considered highly distasteful by *Neoglyphidodon melas*, yet the responses by both *Abudefduf vaigiensis* and *Abudefduf sexfasciatus* were indifferent. *Stephanometra indica* is also highly unpleasant in taste for *Neoglyphidodon melas* but is only weakly deterrent for both *Abudefduf* species. These findings indicate that the taste deterrents of comatulids are not universal in their effectiveness and selectively increase the protection from some fish and do not protect from others that have acquired the ability to overcome deterrent substances. Such evolutionary changes in taste reception are known for animals. As an example, some predatory mammals have become insensitive to sugars after losing their functional genes that confer the ability to express the corresponding taste receptor proteins^61–63^. Most likely, due to the same adaptive transformations, some aquatic animals become able to feed on protected preys, whose deterrence allowed them to avoid other predators^64–66^. It is known that natural taste deterrents are most successfully overcome by specialized consumers that feed on chemically protected organisms. The gustatory deterrents are most effective for omnivorous fish with limited food specialization^57,64,67–71^.

However, the relationship between feeding and susceptibility to gustatory deterrents in fish is apparently not so straightforward and can be related to other features of their biology. Thus, among the fish studied in this report, comatulids possess the strongest aversive taste for *Neoglyphidodon melas* and weaker ones for two *Abudefduf* species. By the mode of life, these three pomacentrids are different. *Neoglyphidodon melas* is a territorial fish, actively protecting its small area from any intruders, especially from conspecifics. It is reported that this fish species feeds mainly on benthic animals, and only within their own territory, whose resources are limited^72–77^. It has been shown that territorial pomacentrids such as *Neoglyphidodon melas* have the most visible effect on the benthic community^78,79^. The comatulids accidentally entering this territory will be most likely exposed to considerable threat, and they especially need effective protection from territorial fish. Interestingly, *Neoglyphidodon melas* can easily feed on soft corals and other sessile marine invertebrates, which also contain taste deterrents and toxic substances^80–83^.

Both *Abudefduf* species are schooling fish that feed on benthic and planktonic organisms^72,73,75,77,84^. One of them, *Abudefduf sexfasciatus*, becomes territorial only during pair formation for spawning. The lack of territoriality expands their resource base, and increases the opportunities for finding food and its selective consumption. In such conditions, even a low deterrence of prey may be enough to encourage fish to search for other food more suitable for their taste.

Earlier, the palatability of twelve species of comatulids was studied using two species of fish, *Canthigaster valentini* and *Chaetodon ephippium*^20^. Three comatulid species, *Himerometra robustipinna, Comaster nobilis* and *Comanthus parvicirrus*, from this study were used also in our experiments. The results coincided for *Himerometra robustipinna*, which demonstrated low deterrence in both studies. For the other two comatulid species, the results of these studies were opposing. The reasons for such a discrepancy may be related to the different methods employed for the evaluation of palatability and with the specific characteristics of the fishes used for the experiments. The other possible reasons are the seasonal, biotopic and geographical variability of taste properties of comatulids^85–92^.

### Relationship between comatulids and their symbionts

Due to their high palatability for fish, comatulid symbionts do not take part in the chemical defence of their host. At the same time, the chemical protection of comatulids is undoubtedly useful for symbionts. In the case of a successful attack, the hunting predator will most likely grasp not only a hiding symbiont but also a fragment of an arm or a pinnula of a comatulid. Such food, consisting of palatable symbiont and inedible, chemically and morphologically protected fragments of the host, is unlikely to be consumed by a predator. It is known that chemically protected prey, caught and refused by fish, usually remains intact and most often uses this opportunity to hide and escape the danger^93–98^. Survival after rejection is also known for diverse preys of land and aerial predators^99–102^. Comparison of the chemical protection of comatulids and their symbionts allows us to consider their association as commensalism, when one of the partners uses the other’s protection as a refuge.

To the best of our knowledge, there are few papers showing an increase the host’s chemical security by symbionts^31,33–35,37^. More often, the symbionts become the beneficiaries. For example, the amphipod *Polycheria antarctica* f. *acanthopoda* forms slit-like depressions in the tunic of the chemically defended colonial ascidian *Distaplia cylindrica*. By hiding in such a shelter and closing the entrance to it, the amphipod is believed to avoid fish consumption^103^. When a potential danger occurs, the Antarctic amphipod *Gondogeneia antarctica* leaves the algae on which it feeds and seeks protection among other chemically protected algae. The other sympatric amphipod *Prostebbingia gracilis* permanently resides among chemically protected algae and tries not to leave this relatively safe shelter^104^. Various algae grow better in the vicinity of chemically protected soft scleractinian and gorgonian corals^105,106^. The hosts seem to attract symbionts not only as a suitable substrate, shelter and food, but also as an opportunity to obtain additional chemical protection and become less vulnerable to predators. We believe that not only comatulids but also other chemically protected hosts from different taxa provide more preferential habitats for symbionts.

## Conclusion

One of the main inferences of our study is that the presence of symbionts stimulates the attacks of omnivorous fish on comatulids, which leads to their injury. Since different species of symbionts have different attractiveness for predators, one can expect a higher incidence of injuries in host individuals inhabited by tasty symbionts, such as *Synalpheus* spp. in our study. Higher incidence of injuries and regeneration can reduce feeding, growth rates, and the reproductive potential of comatulids, as occurs in several other echinoderms^92,107^. Thus, the presence of tasteful symbionts may have significant impacts on the individual fitness and population dynamics of comatulids. Whether symbionts are able to enhance the incidence of comatulid injuries and regulate their abundances remains a topic for future studies.

Like the crinoids, many coral-dwelling animals and plants have a diverse symbiotic fauna and flora. Seemingly similar relationships can be established in many other associations, where symbionts inhabit chemically defended hosts. However, how characteristic the type of relationship we identified is among the comatulids and their symbionts, and how widespread they are in marine communities, especially in coral reefs, remain to be assessed.

## Materials and methods

### Species

**Figure 4:**
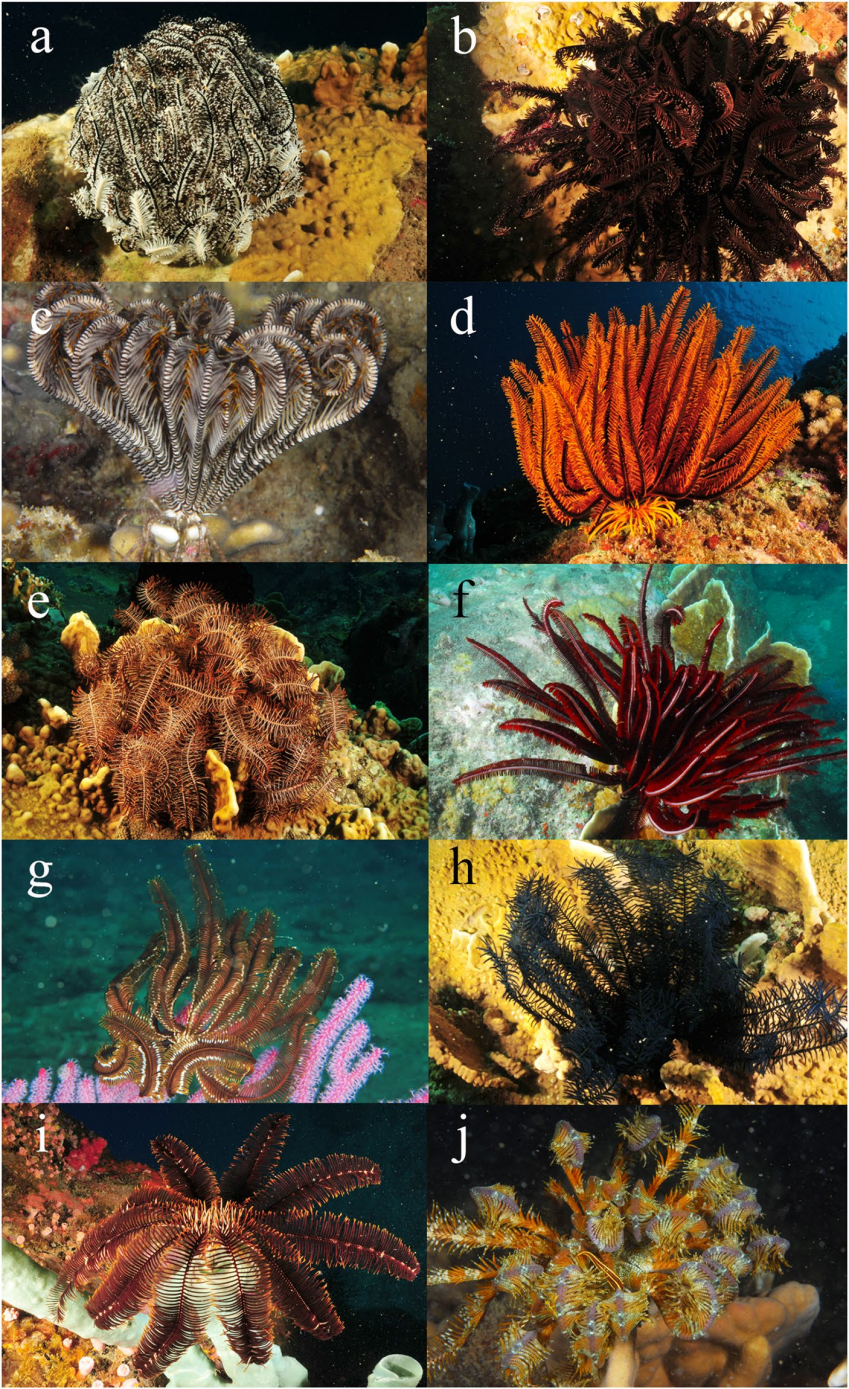
Comatulids employed in experiments: (**a**) *Comaster nobilis*, (**b**) *Clarkcomanthus alternans*, (**c**) *Stephanometra indica*, (**d**) *Anneissia pinguis*, (**e**) *Comanthus gisleni*, (**f**) *Himerometra robustipinna*, (**g**) *Cenometra bella*, (**h**) *Comanthus parvicirrus*, (**i**) *Colobometra perspinosa*, and (**j**) *Lamprometra palmata*. Photo courtesy of O.V. Savinkin.

**Figure 5:**
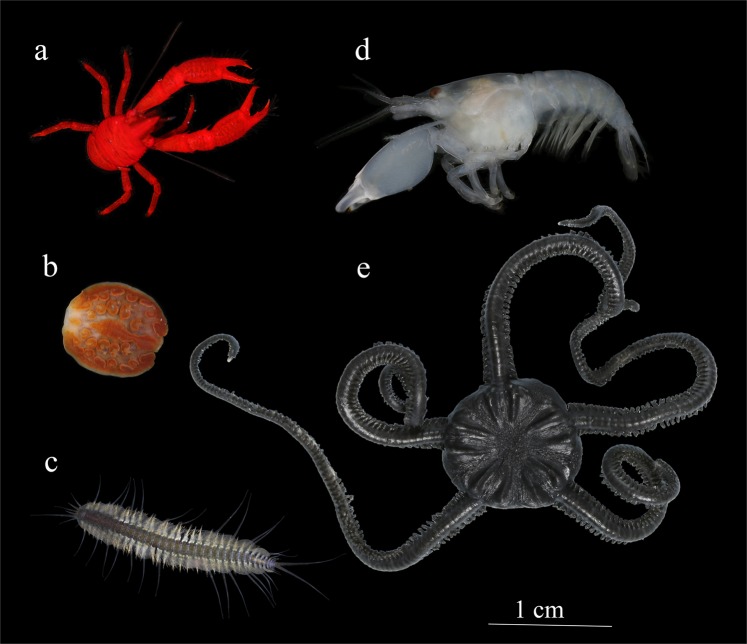
Comatulid symbionts employed in experiments. (**a**) crustacean galatheid *Allogalathea elegans*, (**b**) myzostomid *Notopharyngoides aruensis*, (**c**) polychaete *Paradyte crinoidicola*, (**d**) alpheid shrimp *Synalpheus* sp., and (**e**) ophiuroid *Gymnolophus obscura*.

The field work was conducted at the base of the Coastal Branch of the Russian-Vietnamese Tropical Scientific-Research and Technological Center (Nhatrang city, Vietnam) in 2013–2015. Ten species of comatulids (*Clarckomanthus alternans* (Carpenter, 1881), *Comanthus gisleni* Rowe, Hoggett, Birtles & Vail, 1986*, Comanthus parvicirrus* (Müller, 1841)*, Cenometra bella* (Hartlaub, 1890)*, Colobometra perspinosa* (Carpenter, 1881), *Comaster nobilis* (Carpenter, 1884)*, Himerometra robustipinna* (Carpenter, 1881)*, Lamprometra palmata* (Müller, 1841)*, Anneissia pinguis* (AH Clark, 1909), and *Stephanometra indica* (Smith, 1876), and six species of their symbionts, the polychaete *Paradyte crinoidicola* (Potts, 1910), the myzostomid *Notopharyngoides aruensis* Remscheid, 1918, the galatheid *Allogalathea elegans* (Adams & White, 1848), shrimps *Synalpheus stimpsoni* (de Man, 1888) and *S. demani* Borradaile, 1900 (if specific identification of *Synalpheus* shrimps was not possible, it was marked as *Synalpheus* sp.)*, and* the brittle star *Gymnolophus obscura* (Ljungman, 1867) were employed in experiments (Figs. 4 and 5).

**Figure 6:**
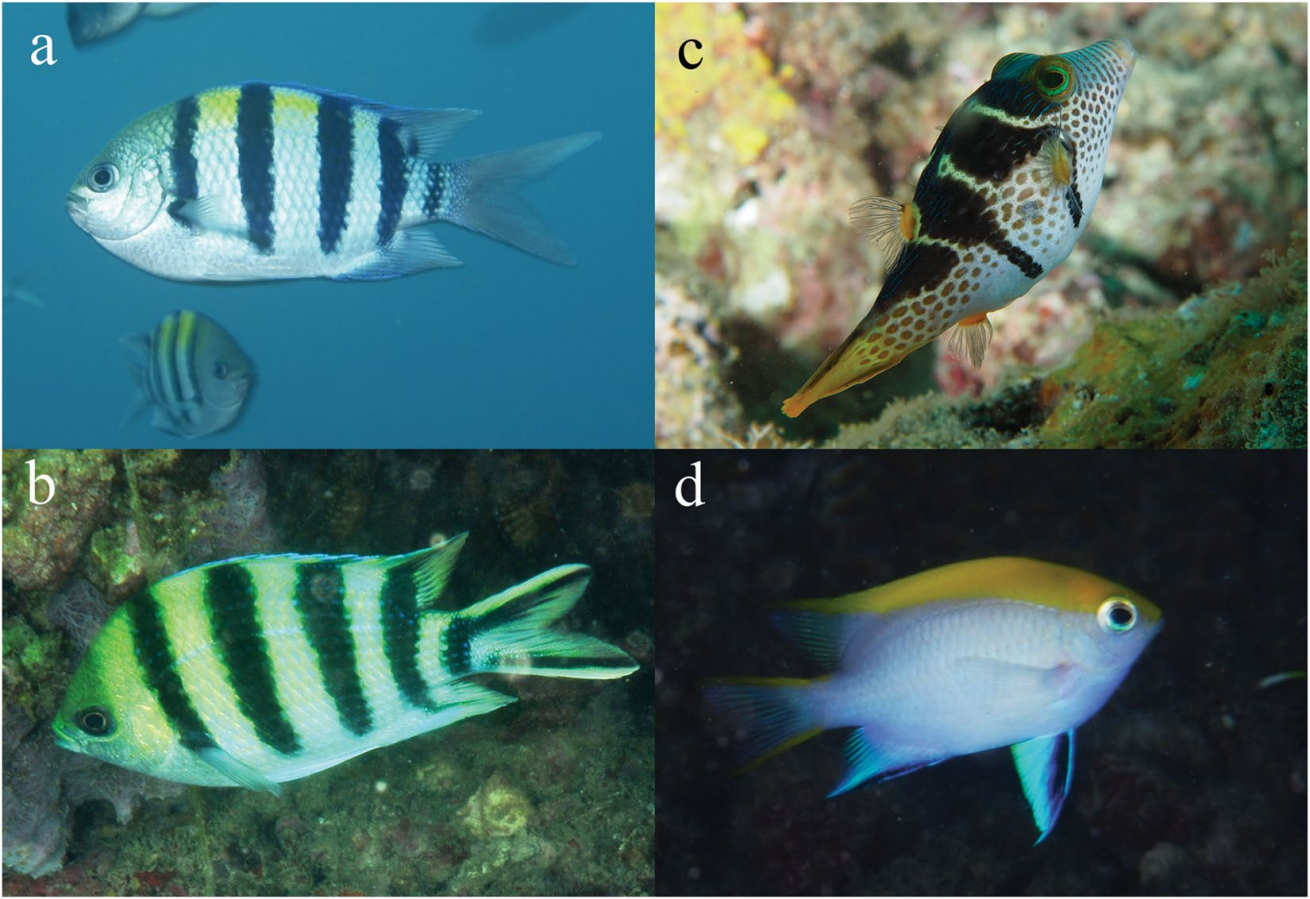
Fish employed in experiments. (**a**) Indo-Pacific sergeant, *Abudefduf vaigiensis*, (**b**) scissortail sergeant, *Abudefduf sexfasciatus*, (**c**) Valentin’s sharpnose puffer, *Canthigaster valentini*, (**d**) black damsel *Neoglyphidodon melas* (**a,b**) Photo courtesy of Yu.V. Deart. (**c,d**) Photo courtesy of O.V. Savinkin.

Assessments of taste attractiveness of extracts were carried out on four species of coral fish similar in size (6–12 cm) and common in the Bay of Nhatrang: Indo-Pacific sergeant, *Abudefduf vaigiensis* (Quoy & Gaimard, 1825) (n = 12), scissortail sergeant, *Abudefduf sexfasciatus* (Lacepède, 1801) (n = 24), black damsel, *Neoglyphidodon melas* (Cuvier, 1830) (n = 24) (all – Pomacentridae), and Valentin’s sharpnose puffer, *Canthigaster valentini* (Bleeker, 1853) (n = 6) (Tetraodontidae) (Fig. 6). This number of used fish is common for experiments where the chemical defense of various hydrobionts against fish is studied (see for review^96,108–110^), and it is in consistence with 3 R principle for bioethics. In many such studies fish, or groups of fish, were used repeatedly^50,51,111^. The main reasons for choosing these fish species were that: i) they are sympatric with comatulids and inhabit the same biotops in coral reefs, ii) they are abundant, and iii) they are generalist consumers and can feed on unprotected comatulids and other invertebrates and plants as well.

### Sample collection

Comatulids and their symbionts were hand-collected by SCUBA divers in Dambay (Tre Is., Nhatrang Bay, Vietnam, South China Sea). Fish were caught with hand nets in the same places as comatulids. Sampling was performed at depths of 2–15 m, largely during the day, but some comatulid specimens were collected at night. Comatulids were gently pulled away from the substrate, immediately placed in individual zip-locked plastic bags, and transported to the laboratory in aerated tanks for further processing.

To obtain symbionts, comatulids were examined with both the naked eye and under a stereo microscope. Additional symbionts were collected by washing the comatulids for 1–2 min in a small container with MgCl_2_·6H_2_O solution (1 part magnesium and 12 parts water, by volume), and the solution was then sieved through a 500-μm mesh. Then, symbionts were placed in clean water for 10–15 min to wash out the magnesium and were sieved again. In comatulids, distal parts of the arms were amputated in 2–3 individuals of each species. Obtained biomaterials were stored at −18 °C for up to 7–12 days until preparation of the water extract for the assay.

### Preparation of pellets

To prepare the extract, samples were homogenized in a ceramic mortar with a small amount of sea water, and after 15 min of extraction, they were centrifuged at 4000 rpm for 20 min at a temperature of +3 °C (Eppendorf Centrifuge 5430 R). The supernatant and an aqueous solution of the dye Ponceau 4 R were added to a hot solution of agar (60–70 °C) prepared in sea water, and the mixture was stirred and poured into a Petri dish. Secondary metabolites which are supposed to be deterrent substances in comatulids^60^ most probably are resistant for such treatment^112–114^. The concentrations of agar and Ponceau 4 R in the gel matrix were 2% and 5 μM, and the concentrations of most extracts were 300 mg/ml (see Table 1). The gel was stored at +5 °C for no more than 3 days. Cylindrical pellets (diameter 1.3 mm, length 4.0 mm) were cut out from the gel with a thin-walled stainless steel tube just before each trial. Control pellets were cut from a gel containing only dye.

### Fish maintenance

After delivery to the laboratory, the fish were kept in common aquariums (150–250 l) for 1–2 weeks. A few days before the experiment, selected healthy fish were individually placed in small aquariums (29 × 25 × 25 cm; 15 l) without gravel and with an aperture in the lid for feeding or presenting experimental pellets. Aquariums were combined into a closed system in which water was pumped from the external biofilter (60 × 45 × 80 cm; 200 l) simultaneously to all aquariums (1.5–1.7 l/min) by an AP5600 pump (Guangdong Zhenhhua Electrical Appliance Co., Ltd; China) and then returned to the filter. The water was aerated before entering the aquariums, and its temperature varied from 28 to 30 °С. Every 3–5 days, half of the water circulating in the system was replaced with fresh sea water.

Acclimation of fish in separate aquaria occurred most rapidly for *Abudefduf vaigiensis* and *Neoglyphidodon melas*. In a few days, they began to willingly grasp any pellets falling into the water and ceased to be afraid or anxious. In the behaviour of *Neoglyphidodon melas*, signs of territoriality quickly became noticeable: they attacked any large moving objects that appeared in the aquarium (net, siphon tube, etc.)*. Canthigaster valentini* also quickly adapted to the new conditions and demonstrated generally calm and phlegmatic behaviour that could turn, similar to *Neoglyphidodon melas*, into aggression. Acclimation of *Abudefduf sexfasciatus* was slow; some individuals remained extremely shy and may have refused to eat for more than a week. Even when they started to pick up food or pellets, they preferred to do this only when the researcher left the area. When the researcher approached or made a sudden movement near the aquarium, the fish easily became frightened, went to the back wall, began to swim and rush nervously, and then sometimes remained motionless at the bottom or at the back wall of the aquarium for a long time (tens of minutes or hours). Most individuals of *Abudefduf sexfasciatus* remained wary even after several weeks spent in the aquarium. Some of them showed fear and panic reactions to the researchers’ actions and a persistent refusal to eat during the entire time they were kept in captivity.

### Experimental design

**Figure 7:**
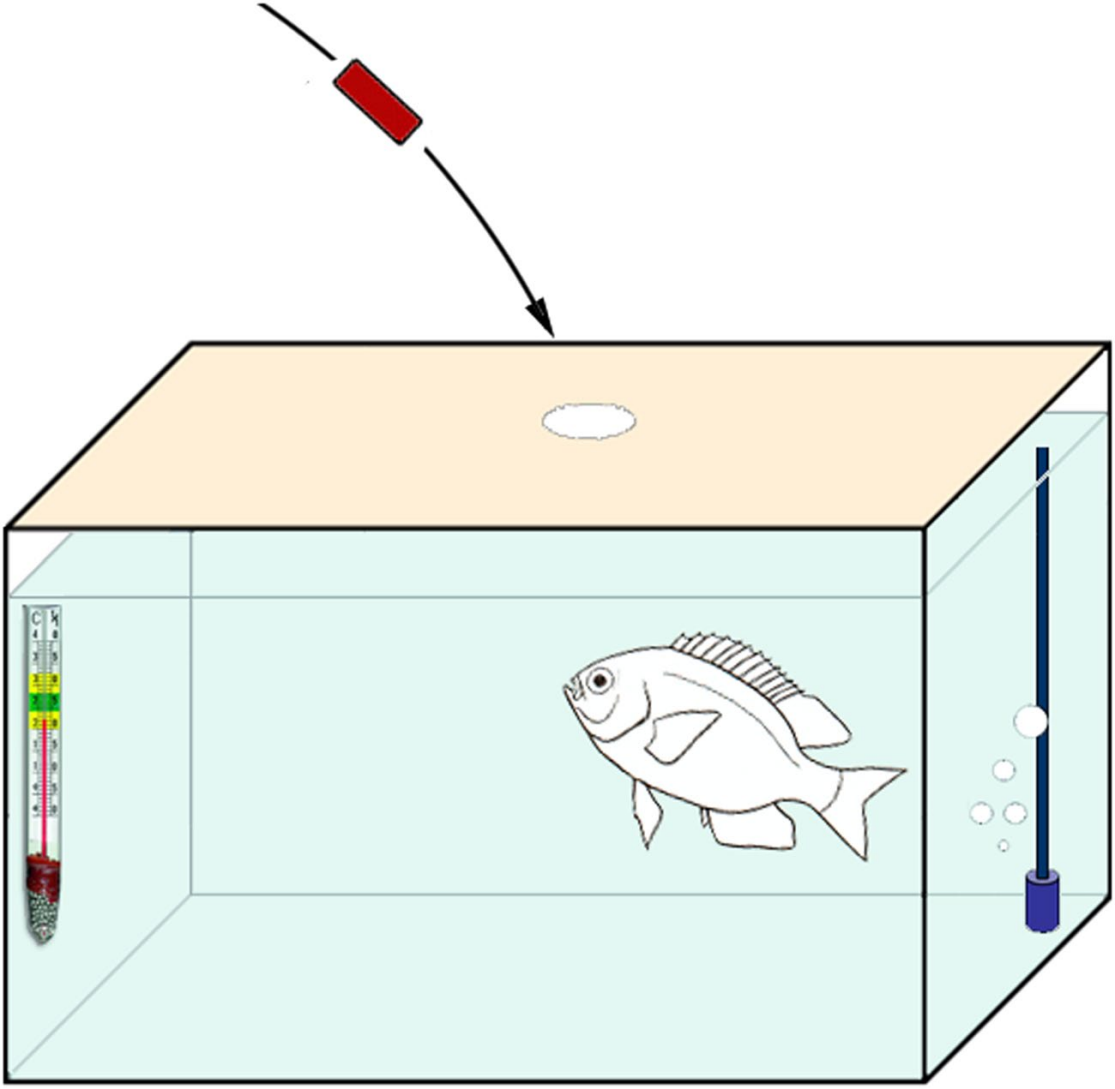
Experimental setup: test tank (15 l) equipped with an aerator and temperature indicator; a single fish first trained to grasp red-coloured, standard-sized pellets is in waiting pose.

Before the experiments, the fish were trained to grasp individually offered food pellets with water extract of shrimp *Penaeus vannamei* Boone, 1931. After completing this training (2–3 days), the fish grasped the presented pellets within 3–5 s of their falling into the water (Fig. 7). The fast grasping as well as the short retention time for oral testing (less than 10 s) prevents diffusing substantial amount of water-soluble substances from pellet to the water column.

In each trial, one pellet with extract of symbionts or their hosts was introduced to the aquarium. All fish expressed their attitude to the taste of a grasped pellet by either its ingestion or rejection and refusal of consumption. The moment of swallowing the pellet by the fish was determined by the restoration of normal breathing movements and the cessation of chewing movements. When demonstrating its final rejection to consume the pellets, a fish would swim away from the rejected pellets and did not show repeated attempts to grasp or even come close to them. In case of final refusal of fish to consume a grasped pellet, the trial was considered to have ended, as with after ingestion.

The ingestion of a pellet or its final rejection is usually preceded by repeated intermediate rejections of the pellet and subsequent rapid re-grasping. Such feeding behaviour is inherent in all four species of fish. A grasped pellet is retained by the fish in the mouth usually from 0.5–0.7 s to 5–7 s. The longest trials lasted no more than 1 min, and most usually ended 20–30 s after the moment when the offered pellet sank into the water. If the pellet was not grasped within 2 min, or its consumption could not be determined due to pellet destruction by fish, the trial was disregarded. The uneaten pellet or its fragments were removed from the aquarium after the end of the trial. Pellets containing extracts of various species were presented to fish in a random order with an interval of 15–20 min. All experiments were performed during the daytime. Every day after the end of the trials, fish were fed to satiation with fresh or fresh-frozen *Penaeus vannamei*.

Experiments to assess the taste attractiveness of comatulids and their symbionts for *Abudefduf vaigiensis* were performed in 2013–2014, and those for *Neoglyphidodon melas*, *Abudefduf sexfasciatus* and *Canthigaster valentini* were performed in 2015. In total, 3641 trials were conducted.

The authors confirm that all methods were carried out in accordance with relevant guidelines and regulations. In addition, the Russian-Vietnamese Tropical Centre approved all experimental protocols with fish and the collection of the samples and animals used in our article.

### Statistical analysis

Chi-squared tests were conducted to detect statistically significant differences in the consumption of flavoured pellets in relation to the controls. To evaluate the effectiveness of the extracts, the index of palatability was calculated using the formula: Ind_pal_ = [(R − C)/(R + C)] 100, where R is the consumption of pellets with extract in percentage, and C is consumption of control pellets in percentage. To evaluate the similarity between the palatability of extracts of comatulids for three species of pomacentrid fish, Abudefduf vaigiensis, Neoglyphidodon melas and Abudefduf sexfasciatus, the Spearman rank correlation coefficient (*r*_*s*_) was calculated. Correlation analysis (non-parametrical Spearman’s rank correlation coefficient) was used to evaluate the similarity between palatability consumption of agar pellets flavoured with extracts of comatulids for Abudefduf vaigiensis, Neoglyphidodon melas and Abudefduf sexfasciatus. All these fish species are closely related (they belong to the same family Pomacentridae) but differ in feeding ecology and behavior. Correlation analysis gives additional estimate how similar is attitude of these species towards taste of comatulids.
